# A Subset of Purposeless Oral Movements Triggered by Dopaminergic Agonists Is Modulated by 5-HT_2C_ Receptors in Rats: Implication of the Subthalamic Nucleus

**DOI:** 10.3390/ijms21228509

**Published:** 2020-11-12

**Authors:** Mélanie Lagière, Marion Bosc, Sara Whitestone, Abdelhamid Benazzouz, Abdeslam Chagraoui, Mark J. Millan, Philippe De Deurwaerdère

**Affiliations:** 1 Centre National de la Recherche Scientifique (Unité Mixte de Recherche 5287), 146 Rue Léo Saignat, 33076 Bordeaux CEDEX, France; lm-melanie@hotmail.fr (M.L.); marionmariecerise@gmail.com (M.B.); sarawhitestone@gmail.com (S.W.); 2 Centre National de la Recherche Scientifique (Unité Mixte de Recherche 5293), 33076 Bordeaux CEDEX, France; abdelhamid.benazzouz@u-bordeaux.fr; 3 Neuronal and Neuroendocrine Differentiation and Communication Laboratory, Institute for Research and Innovation in Biomedicine of Normandy (IRIB), Normandie Univ, UNIROUEN, INSERM, U1239, CHU Rouen, 76000 Rouen, France; abdeslam.chagraoui@univ-rouen.fr; 4 Department of Medical Biochemistry, Rouen University Hospital, 76000 Rouen, France; 5 Institut de Recherche Servier, Center for Therapeutic Innovation in Neuropsychiatry, Croissy/Seine, 78290 Paris, France; mark.john.millan@gmail.com

**Keywords:** single cell extracellular recording, basal ganglia, c-fos, quinpirole, orofacial, electrical stimulation, substantia nigra pars reticulata, 5-HT_2C_ antagonist, anterior cingulate cortex, compulsive disorder

## Abstract

Dopaminergic medication for Parkinson’s disease is associated with troubling dystonia and dyskinesia and, in rodents, dopaminergic agonists likewise induce a variety of orofacial motor responses, certain of which are mimicked by serotonin2C (5-HT_2C_) receptor agonists. However, the neural substrates underlying these communalities and their interrelationship remain unclear. In Sprague-Dawley rats, the dopaminergic agonist, apomorphine (0.03–0.3 mg/kg) and the preferential D2/3 receptor agonist quinpirole (0.2–0.5 mg/kg), induced purposeless oral movements (chewing, jaw tremor, tongue darting). The 5-HT_2C_ receptor antagonist 5-methyl-1-[[2-[(2-methyl-3-pyridyl)oxyl]-5-pyridyl]carbamoyl]-6-trifluoromethylindone (SB 243213) (1 mg/kg) reduced the oral responses elicited by specific doses of both agonists (0.1 mg/kg apomorphine; 0.5 mg/kg quinpirole). After having confirmed that the oral bouts induced by quinpirole 0.5 mg/kg were blocked by another 5-HT_2C_ antagonist (6-chloro-5-methyl-1-[6-(2-methylpiridin-3-yloxy)pyridine-3-yl carbamoyl] indoline (SB 242084), 1 mg/kg), we mapped the changes in neuronal activity in numerous sub-territories of the basal ganglia using c-Fos expression. We found a marked increase of c-Fos expression in the subthalamic nucleus (STN) in combining quinpirole (0.5 mg/kg) with either SB 243213 or SB 242084. In a parallel set of electrophysiological experiments, the same combination of SB 243213/quinpirole produced an irregular pattern of discharge and an increase in the firing rate of STN neurons. Finally, it was shown that upon the electrical stimulation of the anterior cingulate cortex, quinpirole (0.5 mg/kg) increased the response of substantia nigra pars reticulata neurons corresponding to activation of the “hyperdirect” (cortico-subthalamonigral) pathway. This effect of quinpirole was abolished by the two 5-HT_2C_ antagonists. Collectively, these results suggest that induction of orofacial motor responses by D2/3 receptor stimulation involves 5-HT_2C_ receptor-mediated activation of the STN by recruitment of the hyperdirect (cortico-subthalamonigral) pathway.

## 1. Introduction

Orofacial motor activity, which participates in a large variety of behaviors like feeding, facial cleaning, facial expression, and speech, is controlled by multiple neurobiological networks. It is impaired in several neurological and psychiatric conditions, including tic disorders, while antipsychotic and antiparkinsonian drugs can induce acute and chronic oral dystonia and dyskinesia [1,2,3,4,5]. Dopamine (DA) and serotonin (5-HT), notably acting via the serotonin2C (5-HT_2C_) receptor subtype, both regulate the activity of the basal ganglia, a cluster of subcortical brain regions involved in the control of motor behavior [6,7]. The neurobiological substrates of the interaction between 5-HT_2C_ receptors and the dopaminergic system are still unclear. 

The vast majority of DA agonists trigger oral movements in rodents including chewing, jaw tremor, and tongue darting commonly referred to as purposeless oral movements [8,9,10,11,12]. Furthermore, 5-HT_2C_ receptor agonists also induce purposeless oral movements [13,14,15,16,17] suggesting that the purposeless oral movements elicited by DA agonists might be related to or involve a role for 5-HT_2C_ receptors. In line with this possibility, the non-selective 5-HT_2C_ antagonist mianserin reduced purposeless oral movements induced by the D1 receptor agonist SKF-38393, but not by those induced by a low dose (0.2 mg/kg) of the D2 receptor agonist quinpirole [8,10,13]. However, not all observations are consistent with these findings [18,19]. Thus, further examination of the role of 5-HT_2C_ receptors in oral effects of DA agonists is required to clarify the mechanism of interaction and determine their neural substrates. 

The involvement of 5-HT_2C_ receptors in oral responses induced by DA agonists could be expressed at the level of the basal ganglia. Evidence that 5-HT_2C_ receptors preferentially modulate the activity of cognitive/limbic parts of the basal ganglia is suggested by their influence on several readouts: The electrical activity of DA neurons [20,21]; the electrophysiological responses of substantia nigra pars reticulata (SNr) neurons to cortical stimulation of the prefrontal and the anterior cingulate cortex (ACC) but not the motor cortex [22,23]; and c-Fos expression in the striatum and the subthalamic nucleus (STN) [19,24]. A role of these two nuclei has previously been reported for purposeless oral movements or orofacial stereotypies induced by DA and 5-HT_2C_ receptor agonists upon local injection [25,26,27,28]. However, it is presently unknown whether the interaction of 5-HT_2C_ receptors and DA agonists is associated with specific functional effects in the basal ganglia. A possible relationship of these interactions to compulsive motor behaviors in addition to dyskinesias is also worth consideration [29,30].

In the present study, using the selective 5-HT_2C_ antagonists 5-methyl-1-[[2-[(2-methyl-3-pyridyl)oxyl]-5-pyridyl]carbamoyl]-6-trifluoromethylindone (SB 243213) and 6-chloro-5-methyl-1-[6-(2-methylpiridin-3-yloxy)pyridine-3-yl carbamoyl] indoline (SB 242084), we evaluated the participation of 5-HT_2C_ receptors in the induction of purposeless oral movements by the “mixed” D1/D2 dopaminergic agonist, apomorphine, the preferential D2 vs. D1 (D2/3) receptor agonist quinpirole, and the preferential D1 vs. D2 agonist SKF-38393. The behavioral study allowed for selection of the most appropriate conditions for exploration of the neural underpinnings of the interaction between DA agonists and 5-HT_2C_ antagonists employing several complementary approaches: c-Fos expression in the basal ganglia, electrical activity of the STN neurons, and the response of SNr neurons to electrical stimulation of the ACC. The latter method distinguishes between a potential impact on the “hyperdirect” pathway (cortico-subthalamonigral) vs. the “direct” pathway (cortico-striato-nigral, mainly controlled by D1 receptors), and/or the indirect pathway (cortico-striato-subthalamo-nigral, mainly controlled by D2 receptors) [31,32,33].

## 2. Results 

### 2.1. Effect of 5-HT_2C_ Antagonists on Purposeless Oral Movements Induced by DA Agonists

**Figure 1:**
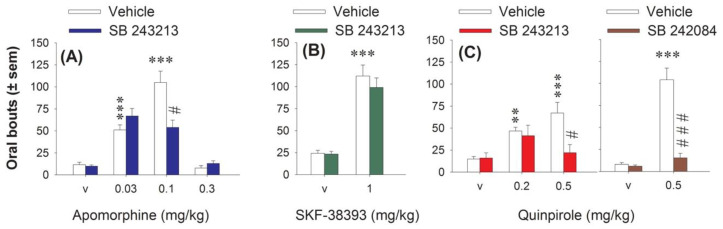
Effect of serotonin2C (5-HT_2C_) antagonists on purposeless oral movements elicited by apomorphine, SKF-38393, and quinpirole. (**A**) interaction of 5-methyl-1-[[2-[(2-methyl-3-pyridyl)oxyl]-5-pyridyl]carbamoyl]-6-trifluoromethylindone (SB 243213) (1 mg/kg intraperitoneal (i.p.)) with apomorphine (0.03, 0.1 and 0.3 mg/kg, i.p.). (**B**) interaction of SB 243213 (1 mg/kg i.p.) with SKF-38393 (3 mg/kg, i.p.). (**C**) interaction of SB 243213 (1 mg/kg i.p. left panel) with quinpirole (Quinp, 0.2 and 0.5 mg/kg, i.p.) and 6-chloro-5-methyl-1-[6-(2-methylpiridin-3-yloxy)pyridine-3-yl carbamoyl] indoline (SB 242084) (1 mg/kg i.p. right panel) with quinpirole (0.5 mg/kg). Each bar of histogram corresponds to the number of movements ± S.E.M. observed for 60 min, after the subcutaneous injection of the D1/D2 agonist, apomorphine or its vehicle, after the i.p. injection of the D1 agonist, SKF-38393 or its vehicle, or after the i.p. injection of the D2 agonist, quinpirole 0.2 and/or 0.5 mg/kg or its vehicle. The injection of the agonist was preceded an hour before by the i.p. injection of SB 243213 or its vehicle (*n* = 6–8 per group). In another experiment, the injection of quinpirole 0.5 mg/kg was also preceded 30 min before by the i.p. injection of the other 5-HT_2C_ antagonist, SB 242084 or its vehicle (*n* = 7–8 per group). ** *p* < 0.01, *** *p* < 0.001 compared to the vehicle group (Fisher’s protected least significant difference (PLSD)). # *p* < 0.05, ### *p* < 0.001 compared to the group treated with the dopamine (DA) agonist alone (Fisher’s PLSD).

Apomorphine, which stimulates all DA receptors, was initially used to link DA transmission to 5-HT_2C_ receptors in the induction of oral movements. Its subcutaneous (s.c.) administration (0.03, 0.1 and 0.3 mg/kg) induced a U-inverted shape dose-response on oral bouts. It dose-dependently increased oral bouts at 0.03 and 0.1, but not 0.3 mg/kg (Figure 1A). Without having any effect by itself, the 5-HT_2C_ antagonist SB 243213 significantly reduced the oral movements induced by apomorphine (three-way ANOVA (“agonist” × “dose of agonist” × “antagonist”) F(2,45) = 9.53, *p* < 0.001) at 0.1 mg/kg only. SB 243213 had no incidence on the oral bouts induced by either 0.03 or 0.3 mg/kg apomorphine. 

The D1 agonist SKF-38393 (3 mg/kg intraperitoneal (i.p.)) also induced purposeless oral movements (Figure 1B), and this effect, at this dose, was previously shown to be blocked by the non-selective 5-HT_2C_ antagonist mianserin [13]. However, the selective 5-HT_2C_ antagonist SB 243213 did not block the oral bouts induced by the D1 agonist (two-way ANOVA (“agonist” × “antagonist”), F(1,31) = 0.36, *p* > 0.05). 

The D2 agonist quinpirole 0.2 and 0.5 mg/kg i.p. dose-dependently increased oral movements (Figure 1C). The 5-HT_2C_ antagonist SB 243213 impaired the effect of quinpirole (three-way ANOVA (“agonist” × “dose of agonist” × “antagonist”), F(2, 38) = 4.59, *p* < 0.05) only at the highest dose of quinpirole (Figure 1C). While this result was in line with the inability of mianserin to block the oral effect elicited by the low dose of quinpirole [8], it was important to confirm with another antagonist that the effect induced by the higher dose of quinpirole was dependent on 5-HT_2C_ receptor. The oral response induced by quinpirole 0.5 mg/kg was higher in this experiment, which has been performed at different periods. Pretreatment with the other selective 5-HT_2C_ antagonist SB 242082 also prevented the oral bouts induced by 0.5 mg/kg quinpirole (F(1,30) = 32.94, *p* < 0.001; Figure 1C). SB 242084, like SB 243213, did not induce oral bouts by itself (Figure 1C).

### 2.2. Effect of 5-HT_2C_ Antagonists on the Number of c-Fos-Immunolabeled Cells Induced by Quinpirole in the Basal Ganglia

**Figure 2:**
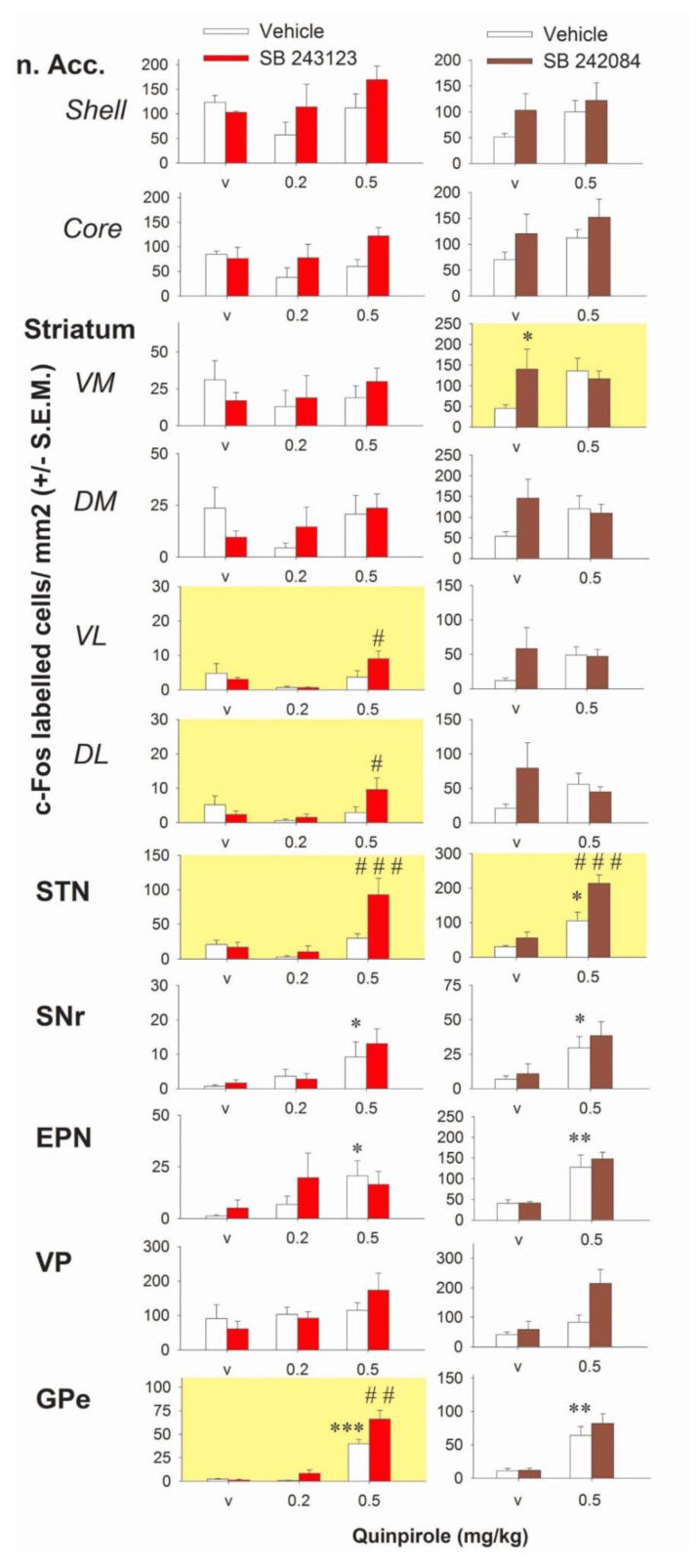
Effect of 5-HT_2C_ antagonism on the number of c-Fos immunolabeled cells by quinpirole in the basal ganglia. The left column corresponds to the interaction of SB 243213 (1 mg/kg i.p.) with quinpirole (Quinp, 0.2 and 0.5 mg/kg, i.p.). The right column corresponds to the interaction of SB 242084 (1 mg/kg i.p.) with quinpirole (0.5 mg/kg). Each bar of the histogram corresponds to the quantitative analysis of the density of c-Fos immunoreactive cells (mean number of cells/mm2 ± S.E.M.) in the nucleus accumbens (n. Acc.) core and shell, the dorsomedial (DM), dorsolateral (DL), ventromedial (VM), and ventrolateral (VL) quadrants of the striatum, the subthalamic nucleus (STN), the substantia nigra pars reticulata (SNr), the entopeduncular nucleus (EPN), the ventral pallidum (VP), and the globus pallidus (GP) of rats. Rats were sacrificed two hours after the intraperitoneal administration of quinpirole or its vehicle (labelled “v” on x-axes). This injection was itself preceded by the i.p. injection of antagonist (colored bars) or its vehicle (white bars), 60 min in the case of SB 243213 and 30 min in the case of SB 242084 (*n* = 6–8 values/group after removing outliers). The yellow background in individual graphs identifies the experiments in which there is a significant interaction between the DA agonist and 5-HT_2C_ antagonism (based on significant two-way or three-way ANOVAs, see results). * *p* < 0.05, ** *p* < 0.01, *** *p* < 0.001 with respect to vehicle-treated rats (Fisher’s PLSD). # *p* < 0.05, ## *p* < 0.01, and ### *p* < 0.001 with respect to the quinpirole (0.5 mg/kg) group (Fisher’s PLSD after significant one-way ANOVA).

Having characterized an interaction between D2 and 5-HT_2C_ receptors on purposeless oral movements, we studied this interaction at the level of the basal ganglia. We used quinpirole over apomorphine because the behavioral data indicated only partial reduction of the effect of apomorphine with the 5-HT_2C_ receptor antagonist. The effects of quinpirole alone or combined with the 5-HT_2C_ antagonists SB 243213 and/or SB 242084 on the number of c-Fos-immunolabeled cells in basal ganglia are reported in Figure 2. Quinpirole gave inconsistent results in the striatum or the nucleus accumbens when compared to vehicle treated rats (*p* > 0.05 for all comparisons; Fisher’s protected least significant difference (PLSD) after one-way ANOVAs in the two experiments concerning quinpirole). The density of cells expressing c-Fos in the striatum and the nucleus accumbens tended to be reduced at 0.2 mg/kg and to be enhanced at 0.5 mg/kg quinpirole when compared to vehicle-treated rats but none of the observed changes reached statistical significance (*p* > 0.05 for all comparisons; Fisher’s PLSD after one-way ANOVAs in the two experiments using quinpirole). Quinpirole at 0.5 but not 0.2 mg/kg, significantly enhanced the number of c-Fos-positive cells in the entopeduncular nucleus (EPN), SNr, globus pallidus (GP), and STN (in one of the two experiments) (Fisher’s PLSD after significant one-way ANOVAs except in the STN of the experiment SB 243213/quinpirole). In the ventral pallidum (VP), quinpirole had no effect on the density of c-Fos-positive cells whatever the dose.

**Figure 3:**
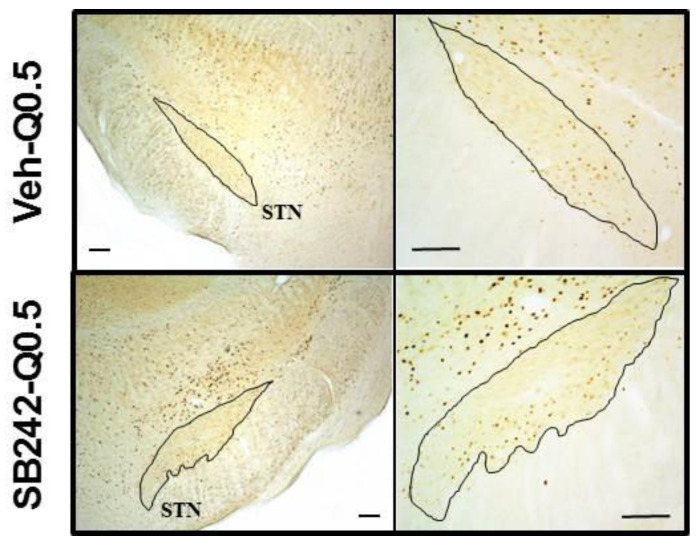
Photomicrographs illustrating c-Fos-like immunoreactive cells at the level of the rat STN. It shows c-Fos labelled cells in a rat receiving quinpirole alone (0.5 mg/kg; Veh-Q0.5; top squares) or in a rat receiving the combination of quinpirole with SB 242084 (1 mg/kg; SB242-Q0.5; bottom squares). SB 242084 enhanced the number of c-Fos-positive cells induced by quinpirole in STN. Scale bar in the left and right squares: 200 and 100 µm, respectively.

SB 243213 unmasked an excitatory effect on the number of c-Fos immunoreactive cells in the presence of 0.5 mg/kg quinpirole in the STN (three way ANOVA (“antagonist” × “agonist” × “dose of agonist”), F(2,32) = 4.56, *p* < 0.05; illustrated in Figure 3) and a modest effect in the ventrolateral (VL) (F(2,33) = 4.45, *p* < 0.05) and the dorsolateral (DL) (F(2,33) = 5.7, *p* < 0.01) striatum. It potentiated the effect of 0.5 mg/kg quinpirole in the external part of the GP (GPe) (F(2,31) = 4.62, *p* < 0.05). SB 243213 did not change the lack of response to 0.2 mg/kg quinpirole on the number of c-Fos-positive cells. SB 243213 did not interact with any dose of quinpirole on c-Fos-positive cells density in the nucleus accumbens and the medial striatum (F(2,33) = 1.12; F(2,33) = 2.09; F(2,33) = 1.71, 1.7, 3.7, and 3.25 in the shell, core, ventromedial (VM), and dorsomedial (DM) of the striatum respectively; not significant for all ANOVAs). Similarly, SB 243213 did not modify the effect of quinpirole in the VP, the EPN, and the SNr (F(2,33) = 1.94, 0.87, and 0.34, respectively; not significant for all ANOVAs). In all brain regions, SB 243213 did not enhance the number of c-Fos-positive cells by itself (Fisher’s PLSD after one-way ANOVAs).

SB 242084 potentiated the effect of 0.5 mg/kg quinpirole on the number of c-Fos-positive cells in the STN (two-way ANOVA (“agonist” × “antagonist”), F(1,27) = 4.34, *p* < 0.05). It interacted also with quinpirole in the VM striatum (F(1,27) = 4.62, *p* < 0.05) but the interpretation of the data is more complex. Quinpirole and SB 242084 both tended to increase the number of c-Fos-positive cells (although it did not reach significance for quinpirole) but the effect obtained by the combination of the two compounds was not additive, and the number of positive cells stayed at the level of SB 242084 alone group. SB 242084 did not unmask effects of quinpirole in striatal territories (F(1,27) = 0.03, 0.19, 3.04, 3.7, and 3.25 in the core, shell DM, DL, and VL of the striatum, respectively; not significant for all ANOVAs). SB 242084 did not significantly modify the effect of quinpirole on the number of c-Fos-positive cells in GP, EPN, and SNr (F(1,27) = 0.63, 0.22, and 0.08; not significant). In the VP, SB 242084 slightly enhanced the density of c-Fos-positive cells induced by quinpirole but this effect did not reach significance (F(1,26) = 2.95; not significant). With the exception of SB 242084 in the VM striatum, SB 242084 increased the density of cells expressing c-Fos by itself, mainly in striatal quadrants, but the effect reached statistical significance only in VM striatum as indicated above.

### 2.3. Effect of Quinpirole/5-HT_2C_ Antagonist on ACC Stimulation-Evoked Responses of SNr Neurons

**Figure 4:**
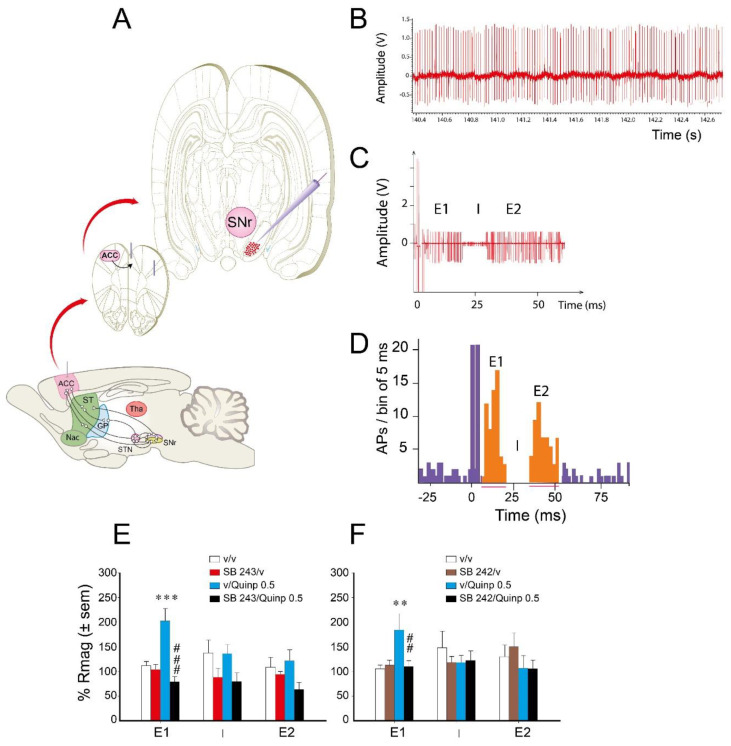
Effect of 5-HT_2C_ antagonism on the cortical responses of SNr neurons influenced by quinpirole. (**A**) schematic representation of cortical stimulation in the anterior cingulate cortex (ACC) and recording sites into the SNr. Each dot in the frontal slides represents a cell responding cells to the ACC stimulation. The responding neurons were located in the medial part of the SNr. The sagittal view of the brain reports the neuronal pathways linking the ACC to the SNr via the subthalamic nucleus, the striatum, and the GPe. (**B**) representative recording trace showing the firing rate of one SNr neuron before any injection (V, volt). (**C**) response of a SNr neuron to 50 ACC electrical stimulations (500 or 700 µA) showing the increased number of action potentials in two periods during the 60 ms following the cortical stimulation and the disappearance of action potentials in-between the two phases of excitatory responses. (**D**) peristimulus time histogram (PSTH, number of action potentials (APs) per bin of 5 ms) representing the overall response of one SNr neuron to 50 cortical stimulations (0.3 Hz). It illustrates three sequential blocs of responses made by an early excitation (E1), an inhibition (I), and a late excitation (E2). The E1 and E2 phase are characterized by an increase of the number of AP per bin which is above the mean number of spikes before stimulation plus 50% (M + 50%). The inhibitory response corresponds to the time interval during which no spike was recorded. (**E**,**F**) effect of the 5-HT_2C_ antagonists SB 243213 (**E**) or SB 242084 (**F**) on the response magnitude (Rmag) of each phase of SNr neuron responses evoked by ACC stimulation with or without quinpirole (Quinp, 0.5 mg/kg). Data correspond to the mean ± S.E.M. of Rmag (in percentage of initial response magnitude, Rmag initial) for E1 (*n* = 7–11 rats per group), I (*n* = 7–11 rats per group), and E2 (*n* = 7–15 rats per group). Data was measured 30 min after intraperitoneal injection of quinpirole or its vehicle. ** *p* < 0.01, *** *p* < 0.001 vs. the vehicle-treated group (Fisher’s PLSD test). ## *p* < 0.01, ### *p* < 0.001 with respect to quinpirole alone group (Fisher’s PLSD test). Of note, no significant difference was observed concerning the basal values of initial Rmags, durations, and latencies between the four groups (not significant one-way ANOVA between values before 5-HT_2C_ antagonist or their vehicle injection). The basal firing rate of SNr neurons was 27.5 ± 1.5 Hz in E, and 22.9 ± 2.15 Hz in F. It did not significantly differ between the different groups before pretreatment injection.

To determine the pathways of the basal ganglia involved in the interaction between 5-HT_2C_ antagonists and quinpirole (0.5 mg/kg), we used single cell extracellular recordings of SNr neurons responding to the electrical stimulation of the ACC (Figure 4A). We recorded 90 neurons with a mean ± S.E.M. discharge frequency of 25.1 ± 2.5 spikes/s and a regular pattern of discharge. ACC electrical stimulation dramatically changed the firing activity of SNr neurons. It occasionally led to the phasic appearance of action potentials during the 60 ms that followed the artifact of stimulation (Figure 4C) in addition to those reflecting tonic firing. The peristimulus time histogram (PSTH) obtained after 50 electrical pulses indicated that ACC electrical stimulation induced a triphasic (47.8%, Figure 4D) or biphasic (52.2%) response of SNr neurons responsive to the stimulation. 

As previously shown ([32,33], Figure 4D), the response of SNr neurons was characterized by an early excitation (E1) (via the cortico-subthalamonigral pathway), an inhibition (I) (via the cortico-striato-nigral pathway), and a late excitation (E2) (via the cortico-striato-pallido-subthalamo-nigral pathway). The biphasic response could have different components (E1 + I, I + E2 or E1 + E2). The physiological response of SNr neurons to a 500 or 700 µA intensity of ACC stimulation was compatible with previous data [23,32,33] concerning the duration and the magnitude of each phase (Table 1).

The main effect induced by quinpirole was observed on the magnitude of the early excitatory E1 phase of the response to ACC stimulation (Figure 4E,F). Specifically, quinpirole enhanced the magnitude of the response corresponding to the hyperdirect pathway. SB 243213, without effect by itself, suppressed the effect of quinpirole on the magnitude of the E1 response (Figure 4E; two-way ANOVA (“agonist” × “antagonist”), F(1,40) = 17.81, *p* < 0.001). No interaction between quinpirole and SB 243213 was observed regarding the I (F(1,33) = 0.097, F(1,33) = 0.007, not significant for the magnitude and duration, respectively) and the E2 (F(1,42) = 2.04, F(1,42) = 0.54, not significant for the magnitude and duration, respectively) responses. Similarly, SB 242084 blocked the effect of quinpirole specifically on the magnitude of the E1 response (Figure 4F; two-way ANOVA (“agonist” × “antagonist”), F(1,28) = 4.82, *p* < 0.05). SB 242084 did not alter the magnitude of E1 by itself (Fisher’s PLSD). Quinpirole and SB 242084 did not modify the duration of the E1 response (F(1,28) = 2.79, not significant), the magnitude and duration of the I (F(1,30) = 1.92, F(1,30) = 4.92, not significant), and the E2 (F(1,39) = 0.2, F(1,39) = 4.03, not significant) responses.

Concomitantly, we measured the basal electrical activity of the SNr neurons that responded to the ACC stimulation. The combination of quinpirole and SB 243213 increased the discharge frequency (two-way ANOVA (“agonist” × “antagonist”), F(1,28) = 7.7, *p* = 0.01) with a maximal effect reached 20 min after quinpirole injection (data not depicted in the graph). Neither quinpirole nor SB 243213 alone altered the spontaneous electrical activity compared to rats receiving vehicles (Fisher’s PLSD). SB 242084 and quinpirole did not significantly change the discharge frequency of SNr neurons at any time point (two-way ANOVA F(1,26) = 0.63, not significant). Neither quinpirole nor SB 242084 alone altered the spontaneous electrical activity.

### 2.4. Effect of SB 243213 and Quinpirole on Basal STN Neuron Activity

**Figure 5:**
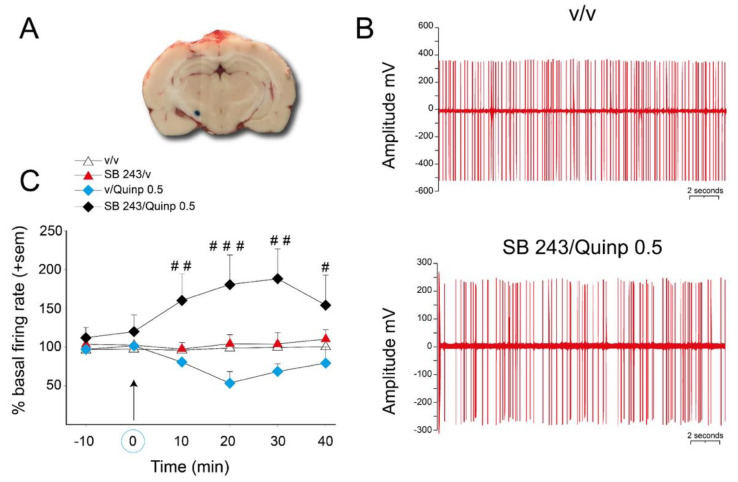
Effect of SB 243213 and quinpirole on the activity of STN neuron activity. (**A**) photomicrograph showing one electrode placement in the STN (blue dot). (**B**) electrophysiological recording of STN neurons receiving saline or the combination of quinpirole and SB243213. Instead of regular discharge, STN neurons receiving the combination of the drugs fired irregularly. (**C**) effect of quinpirole on the discharge frequency of SNr neurons pretreated by SB 243213 (1 mg/kg) or its vehicle. Quinpirole 0.5 mg/kg was injected 60 min after SB 243213 (correspondingly marked on the x-axis and by the arrows). The values indicate the percentage of the basal frequency of discharge ± S.E.M. measured during 40 min after the i.p. injection of quinpirole or its vehicle. # *p* < 0.05, ## *p* < 0.01, and ### *p* < 0.001, Fisher’s PLSD test after significant two-way ANOVA performed on each time point.

To confirm the existence of an interaction between the 5-HT_2C_ antagonist SB 243213 and quinpirole 0.5 mg/kg at the level of the activity of STN neurons, we used extracellular single cell recordings of STN neurons in anesthetized rats (Figure 5). Basal discharge frequency was 10.6 ± 2.1 spikes/s for the recorded neurons, which fired mostly in a regular discharge pattern (Figure 5B, upper action potential traces). The combination of SB 243213 and quinpirole treatments lead to an irregular discharge pattern of STN neurons (Figure 5B, lower action potential traces) with bursts. The mean firing rate (Figure 5C), the number of bursts per second and percent of spikes in bursts (data not depicted in the graph, described below) were dramatically enhanced by the combination of SB 243213 and quinpirole treatments (repeated two-way ANOVA (“agonist” × “antagonist”), F(1,11) = 17.7; 16.3; 16.1 respectively, *p* < 0.001). These effects were already observed at 10 min and reached a maximum at 20 or 30 min (see Figure 5B for the irregularity of the discharge pattern and 5C for the time course of effect on the mean firing rate). More precisely for the burst activity, 30 min after quinpirole injection, the percent of spikes in burst reached 637 ± 100% in the combination group compared to 159 ± 53% in the vehicle group (*p* < 0.001). The number of bursts/s (in percent of basal activity before injections) was also increased to 1276 ± 412 when drugs were combined vs. 169 ± 65 in solvent group (*p* < 0.05). At variance, the number of spikes/burst and the mean duration of the bursts were similar between the groups. The post-hoc test did not reveal any effect of SB 243213 or quinpirole alone at any time point of the time course for the mean firing rate, the number of bursts per second, and percent of spikes in bursts (PLSD Fisher’s test).

## 3. Discussion

The present study provides evidence that 5-HT_2C_ receptors modulate the purposeless oral motor responses induced by the broad-spectrum DA agonist apomorphine and by the D2 (D2/3) agonist quinpirole over a specific range of doses. Importantly, this work unveils an involvement of the STN in this interaction, presumably involving the hyperdirect (cortico-subthalamonigral) pathway of the basal ganglia. Thus, the present data indicate that, via actions expressed in the STN, 5-HT_2C_ receptors modulate discrete subsets of purposeless orofacial responses triggered by dopaminergic receptor agonists and likely reflecting activation of D2-like receptors. These observations are of particular significance since they challenge previous, less extensive studies, whereby it was proposed that 5-HT_2C_ receptors actually control purposeless oral movements induced by D1 receptor stimulation [19].

### 3.1. 5-HT_2C_ Receptors and Purposeless Oral Movements Induced by DA Agonists

In agreement with previous studies [8,34], the mixed D1/D2 agonist apomorphine and the D2 agonist quinpirole enhanced bouts of abnormal orofacial movements. The selective 5-HT_2C_ antagonist SB 243213 reduced or abolished the action of apomorphine and the higher (0.5 mg/kg) dose of quinpirole which, based on previous observations, very likely stimulates D2 receptors [35]. It was important that we corroborated these observations using a further selective 5-HT_2C_ antagonist SB 242084 underpinning the involvement of 5-HT_2C_ receptors in the oral activity induced by a D2 receptor stimulating dose of quinpirole. Moreover, in line with our data with SB 243213, the non-selective 5-HT_2C_ antagonist, mianserin was devoid of effect upon oral bouts elicited by 0.2 mg/kg quinpirole [8]. Thus, based on the present and previous data, the oral purposeless movements induced by quinpirole differentially involve a role of 5-HT_2C_ receptors as a function of dose: Dependent (“high” doses) vs. independent (lower doses), respectively.

Apomorphine’s ability to induce purposeless oral movements was lost at 0.3 mg/kg, which is consistent with previous data indicating that other apomorphine-triggered behaviors, such as yawning or penile erection, are lost as the dose increases [36]. The results with apomorphine were consistent although the reduction of the oral activity by SB 243213 was only partial for the moderate dose of apomorphine. This is likely due to the fact that apomorphine would include oral bouts elicited by stimulation of both D1 and D2 receptor families. Indeed, we found that SB 243213 was devoid of effect on the D1 agonist SKF-38393-induced oral responses. Conversely, mianserin consistently reduced oral responses induced by SKF-38393 (3 mg/kg i.p.) [8,10,25]. These data suggest that the influence of mianserin on SKF-38393 cannot be related to its 5-HT_2C_ antagonist properties, and possibly its blockade of 5-HT_2A_ or α2-adrenergic receptors [37]. This agrees with previous findings showing that the oral activity induced by SKF-38393 and the 5-HT_2C_ agonist meta-chlorophenylpiperazine (m-CPP) was additive [18]. This involvement of 5-HT_2C_ receptors in the purposeless oral movements induced by specific doses of D2 receptor recruiting agonists (permissive or more likely mediating) is fully coherent with the ability of all 5-HT_2C_ agonists tested to induce purposeless oral movements [9,14,16,17,20,22,38].

### 3.2. Quinpirole and 5-HT_2C_ Receptors Interact on STN Neuronal Activity: Focus on the Cortico-STN Hyperdirect Pathway

The question arises as to whether the modulation of purposeless oral movements by the combination of quinpirole and a 5-HT_2C_ receptor antagonist is associated with changes of activity in the basal ganglia, a question addressed by a palette of complementary neurochemical and electrophysiological approaches. Using the expression of the proto-oncogene c-Fos (density of c-Fos-positive cells) as an indirect marker of change of neuronal activity [24], our data reveal that the actions of quinpirole and its interplay with 5-HT_2C_ antagonists is associated with marked changes in the STN. Quinpirole enhanced the number of c-Fos-positive cells in the basal ganglia though to a lesser extent in the STN and striatum [39]. The action of the highest dose of quinpirole was markedly potentiated by both 5-HT_2C_ antagonists only in the STN. An interaction between the higher dose of quinpirole and 5-HT_2C_ antagonists was observed in other loci, notably in a few striatal quadrants and the GP, but the data were not consistent between the two antagonists.

In addition to subtle differences in the reactivity on the density of c-Fos-positive cells in response to the two antagonists, the electrophysiological activity of SNr neurons in the presence of quinpirole was different between SB 243213 (potentiation) and SB 242084 (no interaction). It is interesting to consider why this may be the case. Both antagonists are selective for 5-HT_2C_ receptors [40,41,42], but they differ in regards to the intrinsic activity of their interactions with 5-HT_2C_ receptors, SB 243213 and SB 242084 being characterized as a neutral antagonist and a partial agonist at the Gq phospholipase C pathway, respectively [43,44]. These differences may be related to their distinct influence on c-Fos expression [45,46,47].

Interestingly, we found that the interaction between quinpirole and 5-HT_2C_ antagonists occurs along the hyperdirect pathway. Quinpirole increased the magnitude of the early excitatory response of SNr neurons to stimulation of the ACC, an electrophysiological response that involves the hyperdirect pathway passing through STN neurons [32,33,48,49]. Several authors have emphasized that the hyperdirect pathway, by virtue of the shorter conductance time for cortical information, prevents the activation of competing motor programs and is involved in information selection [31,50]. Conversely, quinpirole did not modify the inhibitory response of SNr neurons to cortical stimulation nor the late excitatory response which reflects recruitment of the D2 receptor-dependent indirect pathway [33]. The lack of influence of quinpirole on the D2 receptor-dependent pathway could be related to the high endogenous inhibitory D2 receptor tone, which is revealed by the excitatory effects of antagonists [51]. Notwithstanding the fact the 5-HT_2C_ receptor agonists WAY163909 or Ro-60-0175 facilitated the appearance of the late excitatory response of SNr neurons to the cortical stimulation of the ACC or the prefrontal cortex [22,23], no interaction between 5-HT_2C_ receptor antagonists and quinpirole was observed on the response of the indirect pathway. By contrast to D2 receptors then, there may only be a very low tone of 5-HT_2C_ receptors expressed upon SNr neurons upon cortical stimulation. However, it should not be forgotten that these observations were made under anesthesia.

By monitoring the electrophysiological activity of STN neurons in a separate experiment, we report that the combination of quinpirole and the 5-HT_2C_ antagonist SB 243213 excited STN neuronal activity, even though quinpirole by itself tended to reduce STN neuronal activity, as previously reported [52]. The firing pattern of STN neurons was characterized by the irregularity of the discharge and the burst activity when drugs were combined. Thus, the combination of quinpirole/5-HT_2C_ receptor blockade enhanced STN neuronal activity, corroborating the data obtained with c-Fos. Both the irregularity and the increase in STN neuronal activity induced by the combination of drugs could account for blunting of the quinpirole-induced enhancement of the magnitude of the early excitatory response of SNr neurons to ACC electrical stimulation.

### 3.3. Correlation between the Orofacial Control Exerted by 5-HT_2C_ Receptors and the Activity of the STN

A role for the STN in expression of the responses mediated by 5-HT_2C_ receptors or induced by DA agonists themselves is not unforeseen. The STN is known to be involved in the purposeless oral movements induced by peripheral administration of the non-selective 5-HT_2C_ receptor agonist m-CPP [25], an effect reproduced upon its intra-STN injection [24,25]. The injection of DA agonists into the STN is also known to induce those oral movements [26]. Moreover, diverse in vitro and in vivo data show that non-selective 5-HT_2C_ receptor agonists such as 5-HT, m-CPP, and Ro-60-0175 enhance the electrical activity of STN neurons [53,54,55,56,57] and c-Fos-positive cell density in the STN [22,24]. Bearing in mind this evidence, additional data are needed to more directly establish the functional links between behavioral effects of dopaminergic and 5-HT_2C_ ligands and the modification of STN neuronal activity [23,46,58]. In particular, the effect of 5-HT_2C_ antagonists on c-Fos both alone [45,46,47] and in the presence of quinpirole (this paper) suggests that 5-HT_2C_ receptors can also reduce STN neuron activity. We cannot exclude the possibility that the reduction of quinpirole-evoked abnormal oral movements by the 5-HT_2C_ antagonists and the associated increase in STN neuronal activity are two distinct, congruent mechanisms. Moreover, further study will be required to determine whether the interaction between quinpirole and 5-HT_2C_ antagonists specifically occurs within the STN itself.

Overall these behavioral observations extend the existing data and indicate that only a few motor “building blocks” of orofacial function regulated by DA receptors [12] are sensitive to modulation by 5-HT_2C_ receptor blockade. It is postulated that 5-HT_2C_ receptors intersect with DA transmission for the expression of only restricted domains of orofacial behavior with an impulsive/compulsive dimension. Oral bouts induced by 5-HT_2C_ agonists have been suggested to correspond to compulsive drives or undefined tic responses rather than dystonia or dyskinesia [14,29]. Furthermore, 5-HT_2C_ antagonists reduce compulsive responses [59], notably by acting at the level of the orbitofrontal cortex [60], and promote impulsive responses [61,62,63]. Quinpirole has been also shown to induce tic-like responses and compulsive responses after subchronic/chronic administration in rodents [30,64,65,66], an effect implicating changes of activity of striatal and fronto-subcortical regions [66,67]. In this light, it is interesting that high frequency stimulation of the STN or its inactivation reduces quinpirole-induced compulsive checking behavior in rats [68,69]. In humans, STN neuronal firing rate has been reported to be reduced with an altered pattern of discharge in obsessive compulsive disorders (OCD) patients while the high frequency stimulation of STN reduced OCD symptoms [70]. The higher responsiveness of STN neurons to phasic cortical inputs in the presence of increased dopaminergic tone could be involved in the altered connectivity within the basal ganglia described in OCD patients [71]. Additional data are needed to determine if the D2/5-HT_2C_ receptor interaction we report at the level of STN participates in the establishment of compulsive responses induced by quinpirole upon its chronic administration in rats and OCD in humans.

## 4. Materials and Methods

### 4.1. Animals

Male Sprague-Dawley rats (Dépré breeding center, Saint Doulchard, France) weighing 300–400 g were used. Animals were kept at constant room temperature (21 ± 2 °C) and relative humidity (60%) with a 12-light/dark cycle (dark from 8 p.m.) and had free access to water and food. All animals use procedures conformed to International European Ethical Standards (86/609-EEC) and the French National Committee (décret 2001-464) for the care and use of laboratory animals. Furthermore, the procedures were approved by the Ethical Committee of Centre National de la Recherche Scientifique, Région Aquitaine-Limousin, and the University (N°50120130-A, 2 May 2013). All efforts were made to minimize animal suffering and to reduce the number of animals used.

### 4.2. Behavioral Testing

In order to habituate rats to the procedures of behavioral testing, rats were occasionally moved to the behavioral room during the week preceding the day of the experiment. The behavioral testing was performed by placing each rat in clear square plastic chambers (12″ by 12″ by 18″ height). Before an adaptation period, each animal received an intraperitoneal administration of 5-HT_2C_ antagonists or its vehicle (pretreatment). Thereafter, rats received an injection of one of the diverse DA agonists studied (treatment) and were immediately and continuously observed for 60 min in the testing cage for bouts of oral movements.

Only vacuous chewing, jaw tremors, and tongue protrusion which occurred without any reference to an evident physical target were considered as purposeless oral movements. Moreover, an interval of two seconds without oral activity was required to consider the oral bout fully terminated. Neither the duration of each oral bout nor the number of deflections in a bout were measured [16,24,72]. Oral activity occurring with feeding or licking was not counted. After the one-hour period of behavioral testing, rats were returned to their home cage. The oral bouts were counted by two experimenters unaware of the pharmacological treatment.

### 4.3. Immunohistochemistry

Two hours after the injection of quinpirole, rats were deeply anesthetized with urethane hydrochloride (1.5%) and perfused transcardially with 0.01 M phosphate-buffered saline (PBS, pH 7.4, 37 °C) followed by ice-cold 4% paraformaldehyde in 0.1 M sodium phosphate buffer (PB). Brains were removed, postfixed for 12 h in the same fixative (4 °C), placed in PBS 0.1 M containing 0.03% sodium azide, and stored at 4 °C. Forebrains were cut at 50 µm on the coronal plane on a vibratome (VT1000S, Leica Instruments, Nanterre, France). Sections through the striatum, nucleus accumbens (n. Acc.), VP, GP, EPN, STN, and SNr were collected in PBS (0.1 M, 4 °C) containing 0.03% sodium azide, and stored at 4 °C pending c-Fos immunohistochemistry. After three washes in PBS 0.1 M (pH 7.4) at room temperature, sections were incubated (1 h) in PBS (0.1 M, pH 7.4) containing 1% bovine albumin serum (BSA) with 0.3% triton. Sections were transferred to PBS (0.1 M, 0.3% triton, pH 7.4) containing anti-Fos rabbit polyclonal antibodies (1:8000; SC-52, Santa-Cruz Biotechnology, Dallas, TX, USA) for 40–44 h at 4 °C. After 3 washes in 0.1 M PBS (10 min each), sections were incubated for 2 h in PBS (0.1 M, 0.3% triton, pH 7.4) containing biotinylated goat anti-rabbit IgG (1:200, Amersham Biosciences, Buckinghamshire, UK). After washing (3 times in 0.1 M PBS), sections were incubated in avidin-biotin-peroxidase complex (2 h; ABC Vectastain Elite Kit, Vector laboratories, Burlingame, CA, USA, distributed by Biovalley S.A., Strasbourg, France) for subsequent staining with 3,3′-diaminobenzidine (0.05% w/v; Sigma-Aldrich, Saint-Quentin Fallavier, France) in 0.05 M Tris buffer saline (pH 7.6) containing 0.003% H_2_O_2_ (Sigma). Sections were finally washed three times in 0.05 M Tris saline buffer (pH 7.6). After processing, tissue sections were mounted onto gelatin-alum-coated slides, dehydrated in ascending concentration of ethanol, and coverslipped with Eukitt mounting medium (Calibrated Instruments, Hawthorne, NY, USA).

### 4.4. Surgeries

In electrophysiological experiments, animals were anesthetized with urethane hydrochloride (1.5 g/kg i.p., supplemented by 50 mg/kg i.p. injections, Sigma-Aldrich, France) and fixed in a conventional stereotaxic apparatus (Horsley Clarke apparatus, Unimécanique, Epinay sur Seine, France). Body temperature was monitored with a rectal probe and maintained at 37 °C with a homeothermic warming blanket (model 50-7061, Harvard Apparatus, Les Ulis, France).

### 4.5. Electrical Stimulation of the Anterior Cingulate Cortex

Electrical stimulation of the anterior cingulate cortex (ACC: [A]: 3.2; [L]: 1.0 from the bregma; [H]: 2.0 mm from the cortical surface), ipsilateral to the recording SNr site, was performed through a bipolar stainless steel electrode (SNEX-200, distance between the 2 poles: 100 μm) positioned stereotaxically according to the atlas of Paxinos and Watson (1998). Electrical stimuli were generated from a constant current isolated stimulator (DS3, Digitimer Ltd., TEM SEGA, Pessac, France) connected via a MacLab interface (MacLab 4s ADInstruments, Cambridge Electronic Design, Cambridge, UK) to program stimulations set on a computer (Scope 3.6 software; Cambridge Electronic Design Ltd.). Stimulation consisted of 50 monopolar pulses of a width of 0.6 ms and 500 or 700 μA intensity delivered at a frequency of 0.3 Hz.

### 4.6. Extracellular Single Unit Recordings of SNr and STN Neurons

Single-unit activity of SNr or STN neurons was recorded extracellularly using glass micropipettes filled with 2% pontamine sky blue dissolved in a 0.6 M sodium chloride solution (8–15 MΩ). Initial stereotaxic lowering of the glass micropipette was set according to the rat brain atlas of Paxinos and Watson (1998) at the following coordinates for the STN: [A], −3.8, [L], 2.5 mm and the SNr; [A], −5.3 and [L], 1.8 mm, with respect to the bregma of the animal [73]. The micropipette was lowered at [V]: 6–8 mm for the STN, and the profile of the neuronal discharge was observed before recording as previously reported [74,75,76]. The upper border was determined by identifying slower firing hypothalamic neurons. The upper border of the SNr was determined by identifying dopaminergic neurons of the substantia nigra pars compacta (7–8 mm under the cortical surface) recognizable by their long action potential (>4 ms), low to moderate frequency of discharge (3–8 Hz), and the ascending phase of the action potential [77]. Below this dopaminergic layer, SNr neurons were identified as non-dopaminergic by their classically defined electrophysiological characteristics: Thin duration of action potentials (<2 ms width) and their ability to present high-frequency discharge (>10 Hz) without a decrease in the spike amplitude [32,77]. All the experiments with the electrophysiological recordings of SNr neurons have been performed with the concomitant electrode of stimulation placed in the ACC, and all sampled neurons were sensitive to the electrical stimulation of ACC.

In case of the SNr experiments, action potentials were amplified with a differential preamplifier (GRASS P15F, West Warwick, RI, USA) and a differential amplifier (AM systems 1700, Sydney, Australia). The signal was displayed on an oscilloscope and registered on a computer (Spike 2 software; Cambridge Electronic Design Ltd.). Spikes were separated from noise using a window discriminator (model 121, World Precision Instruments, Hitchin, UK) and sampled online (Spike 2 software) by a computer connected to the CED 1401 interface. In the case of the STN experiments, a different electrophysiological set up was used. Extracellular neuronal activity was amplified, band-pass filtered (300–3000 Hz) using a preamplifier (Neurolog, Digitimer, Welwyn Garden City, UK), and transferred via a Powerlab interface (AD Instruments, Charlotte, NC, USA) to a computer equipped with Chart 5 software (AD Instruments). In all cases (SNr and STN), at the end of each session, the recording site was marked by electrophoretic injection (Iso DAM80, World Precision Instruments, Hertfordshire, UK) of pontamine sky blue after recording the last neuron of the last trajectory through the micropipette at a negative current of 20 µA for 10 min. After completion of the experiments, animals were sacrificed, the brains removed, frozen in isopentane at −45 °C, and stored at −80 °C. Fresh-frozen brains were cut with a cryostat into 20 μm coronal sections.

### 4.7. Drugs

The 5-HT_2C_ antagonists, SB 243213 and SB 242084 (Mark Millan, Servier Paris, France) dissolved in a mixture of NaCl 0.9% containing hydroxypropyl-β-cyclodextrin (8% by weight) plus citric acid (25 mM), were administered i.p. in a volume of 2 mL/kg body weight. The DA agonist apomorphine (Aguettant, Lyon, France) was prepared in saline containing 0.1% ascorbic acid and injected subcutaneously in a volume of 1 mL/kg body weight. The D2 and D1 receptor agonists, quinpirole (quinpirole hydrochloride) and SKF 38393 (SKF 38393 hydrochloride), respectively, were dissolved in isotonic saline solution (0.9%) and injected i.p. as the free base in a volume of 1 mL/kg.

Apomorphine is a mixed D1/D2 receptor agonist [78] which was used to grossly appreciate the involvement of 5-HT_2C_ receptors in purposeless oral movements over a wide range of doses (0.03, 0.1, and 0.3 mg/kg) [36]. The doses of quinpirole (0.2 and 0.5 mg/kg) were chosen on the basis that low doses (0.1 to 0.25 mg/kg) are usually associated with chewing and motor disturbances [10,36], while higher doses could trigger different nature of behaviors as apomorphine [36] with compulsive dimension and beyond [30,68]. The dose of SKF-38393 (3 mg/kg) was used to trigger purposeless oral movements that have been shown to be reduced by the non-selective 5-HT_2C_ antagonist mianserin [9,10]. The doses of 5-HT_2C_ antagonists, calculated as the free base, were chosen on the basis of previous studies to keep both selectivity and efficiency toward 5-HT_2C_ and DA receptors [40,41,42,79,80]. The administration time was chosen on the basis of their pharmacokinetic properties such that they were at their pharmacodynamic maximums [43,45]. In each experimental group, animals received either drug or their appropriate vehicle.

### 4.8. Data Analysis

#### 4.8.1. Fos Immunohistochemistry

According to the procedure previously described [24,45], three sections per region were analyzed. Sections were observed under a light microscope (Axioscope 9500, Carl Zeiss France S.A.S., Marly le Roi, France) equipped with 5×, 10×, or 20× bright field objectives. The light microscope was surmounted by a video camera (Sony DXP 950P, Carl Zeiss France S.A.S. France) and acquired images were analyzed using Mercator software (Exploranova, La Rochelle, France). Prior to quantitative analysis, sections were viewed at low magnification and anatomical regions were outlined based on a rat brain stereotaxic atlas [73]. The approximate anterior-posterior coordinates within which the sections were selected for each brain region were as follows (in mm from interaural line): Core and shell of the n. Acc.: 11–10.2; striatum: 9.8–8.8; VP: 9.2–8.6; GP: 8.2–7.7; EPN: 6.7–6.2; STN: 5.4–4.8; and SNr: 3.7–3.2. The striatum was analyzed in four quadrants corresponding to the dorsomedial, ventromedial, dorsolateral, and ventrolateral striatum at the level of the central striatum. The intensity of c-Fos immunoreactivity, although variable, was easily distinguishable from background labelling. The distribution of immunoreactive cells was examined and counted on each side of the brain, and the data obtained in both sides of each section were pooled. Then, data in the three sections per structure were averaged for each rat and the mean ± S.E.M. of these values (c-Fos-positive cells/mm^2^) was calculated for each group (8 rats per group). The analyses of c-Fos expression were performed by experimenters unaware of the treatment groups and were counter-expertized.

#### 4.8.2. Electrophysiological Experiments

Basal frequency of the discharge of SNr neurons responding to ACC stimulation was determined before drug injection. In the case of the STN (not characterized with respect to ACC stimulation), the basal frequency of discharge was taken after a few minutes of sampling. The frequency of discharge after the drug injections were calculated in percentage of the frequency of basal discharge before pretreatment (±S.E.M.), for each experimental group. Moreover, additional parameters were included using Neuroexplorer (v3.266), Spike 2, and MATLAB (v6.5): Mean frequency, mean interspike interval, percent of spikes in bursts, number of bursts, bursts per second, percent of spikes in bursts, mean burst duration, and the mean amount of the spikes in bursts [74,76].

Response to cortical stimulation: Peristimulus-time histograms were generated from 50 stimulation trials (500 µA or 700 µA, 0.3 Hz) using 5 ms bins. The criterion used to establish the existence of an excitatory response corresponded to the mean of the number of spikes recorded during the 500 ms preceding the onset of cortical stimulation plus two times the standard deviation (SD) of this mean. An inhibition was considered as a period during which the number of spikes was below 70% of the mean of the number of spikes recorded during the 500 ms period preceding the stimulation [22,81]. 

Excitatory and inhibitory response magnitudes (Rmags) were normalized for different levels of baseline activity, allowing for comparison of drug effects on evoked responses independent of effects on baseline activity [81]. The response magnitudes for excitation were calculated with the following equation: Excitation Rmag = (counts in excitatory epoch) − (mean counts per baseline bin × number of bins in excitatory epoch). The response magnitudes for inhibition were calculated as follows: Inhibition Rmag = (counts in inhibitory epoch) − (mean counts per baseline bin × number of bins in inhibitory epoch). Therefore, one Rmag was obtained for each phase of the triphasic response to cortical stimulation.

Thereafter, in order to determine the duration of each phase of the response, PSTHs were generated from 50 stimulation trials using 1 ms bins for each intensity of stimulation. In this case, as previously described [32], the criterion used to establish the existence of an excitatory response was the increase of >50% in the number of spikes as compared with the pre-stimulus frequency. The duration of an inhibitory response corresponded to the time interval during which no spike was recorded. The duration of each phase was measured for the initial response (before any administration) and then 30 min after the DA agonist quinpirole or its vehicle injection.

### 4.9. Pharmacological Treatment and Experimental Design

In the behavioral experiments, SB 243213 (1 mg/kg i.p.) or its vehicle was administered one hour before the DA agonists apomorphine (0.03, 0.1, or 0.3 mg/kg s.c.), SKF 38393 (3 mg/kg i.p.), or quinpirole (0.2 or 0.5 mg/kg, i.p.). The groups of experiments for each agonist were done separately and independently. In all cases, purposeless oral movements had been observed for one hour immediately after the injection of the agonists or their vehicle. In the case of apomorphine at different doses, SB 243213 and/or their vehicles were injected in a counterbalanced order over a 3 weeks period (4 days interval between injections), such that, at the completion of testing, each rat had received all combinations of drugs in a random order (*n* = 8 rats per group).

In the other sets of behavioral experiments (SB 243213 in the presence of SKF-38393; SB 243213 in the presence of quinpirole 0.2 and 0.5 mg/kg; and the 5-HT_2C_ antagonist SB 242084 in the presence of quinpirole 0.5 mg/kg), rats received only one pharmacological treatment (*n* = 8 in each of the four groups). Indeed, rats were sacrificed 2 h after the injection of the agonist in order to proceed to the c-Fos expression immunohistochemistry experiment. At variance with SB 243213, SB 242084 (1 mg/kg, i.p.), or its vehicle was administered 30 min before quinpirole injection (0.5 mg/kg, i.p.).

The electrophysiological experiments were done according to previous published papers with some modifications [22,23]. After identifying a stable baseline frequency of discharge of a SNr neuron, 50 cortical stimulations of the ACC at 500 or 700 µA (0.3 Hz) were delivered to determine the existence of a triphasic or diphasic response. This procedure was performed before the injection of the 5-HT_2C_ antagonists or their vehicle and the values accounted for the initial response (see data analysis). The impact of pharmacological treatment on the initial response of each SNr neuron was studied 30 min after the i.p. administration of the DA agonist quinpirole (0.5 mg/kg, i.p.) or its vehicle.

The frequency of the discharge of SNr neurons was monitored every 10 min during 30 min after quinpirole injection (at that time, the protocol of ACC stimulation started; see above). The frequency of discharge of STN neurons was monitored every 10 min during 40 min after injection of quinpirole.

### 4.10. Statistical Analysis

Results for behavioral parameters are expressed as the mean ± S.E.M. of events recorded for one hour. Results for c-Fos experiments are expressed as the mean ± S.E.M. of immunoreactive cells per mm2 for each structure. In electrophysiological experiments, two parameters have been considered: The frequency of discharge and the response (Rmag and duration) to electrical stimulation. The frequency of discharge of SNr and STN neurons is expressed for each time point of the time course as the percentage of the basal firing rate (in Hz). Data correspond to the mean ± S.E.M. values of the percentage obtained in each experimental group. In the case of cortical stimulation, Rmags and durations were calculated 30 min after quinpirole or its vehicle injection. Rmags were expressed as the percentage of the initial Rmags obtained before any pharmacological treatment. Duration was expressed in ms. Data correspond to the mean ± S.E.M. values in each experimental group. 

The analysis of behavioral, immunohistochemical, and electrophysiological results was done for each studied parameter (number of purposeless oral movements, density of c-Fos-immunolabeled cells, amplitude and duration of each phase, and frequency of discharge) using ANOVAs (“Antagonist” × “agonist” and whenever relevant, “doses of agonist”). When a significant interaction was found (*p* < 0.05), the ANOVAs were followed by a one-way ANOVA (group as the discriminant factor) and by the Fisher’s PLSD procedure as a post-hoc test to allow for multiple comparisons between groups. When the multiple-way ANOVA did not reach significance (no interaction between the factors “agonist” and “antagonist”, we nonetheless performed a one-way ANOVA using group as the main factor and followed by the PLSD test if significant to determine whether the group “agonist” or “antagonist” had an effect on their own. All statistical analyses were done with the statistical software Sigmaplot 11.0.

## 5. Conclusions

Numerous studies have reported that 5-HT_2C_ receptors interact with DA transmission in the brain [40,82,83,84,85,86,87]. The present data support and extend these observations in demonstrating for the first time that the STN fulfills a key role in expression of interactions between 5-HT_2C_ and D2 receptors in suggesting a role of the hyperdirect (cortical-STN-SNr) pathway in the induction of repetitive oral behaviors. The observations of the underlying neural substrates can be related to studies suggesting a role for 5-HT_2C_ receptors in the dyskinesia that clinically emerges following long-term neuroleptic or L-DOPA administration [88,89,90,91]. Finally, the present observations also provide insights into the neural circuits involved in D2 and 5-HT_2C_ receptors related tic-like behaviors and compulsive drives.

## Figures and Tables

**Table 1:** Characteristics of the triphasic response of SNr neurons evoked by ACC electrical stimulation. The electrical stimulation (500 µA or 700 µA, 0.3 Hz) of the ACC induced triphasic or biphasic responses on SNr neurons. The data reported in the table corresponds to the magnitude and duration of the early excitation E1 (*n* = 72), the inhibition I (*n* = 68), and the late excitation E2 (*n* = 83) for a total of 90 sampled SNr neurons exhibiting biphasic or triphasic responses. Results for magnitude are given in Rmag ± S.E.M. (see methods for calculation). The duration of each phase is given in ms ± S.E.M.

	Early Excitation E1	Inhibition I	Late Excitation E2
initial Rmag	16.6 ± 1.1	−15.3 ± 1.4	33.0 ± 2.7
Duration (ms)	8.1 ± 0.5	12.1 ± 0.8	12.7 ± 0.9

## Abbreviations

5-HT	serotonin
5-HT_2C_ receptors	Serotonin_2C_ receptors
DA	Dopamine
SNr	substantia nigra pars reticulata
STN	subthalamic nucleus
NAc	nucleus accumbens
DM	dorsomedial
DL	dorsolateral
VM	ventromedial
VL	ventrolateral
EPN	entopeduncular nucleus
GP	globus pallidus
ACC	anterior Cingular Cortex
VP	ventral pallidum
OCD	obsessive compulsive disorder
Rmag	response magnitude
PSTH	Peristimulus time histogram
PLSD	protected least significant difference
